# BHT-QAOA: The Generalization of Quantum Approximate Optimization Algorithm to Solve Arbitrary Boolean Problems as Hamiltonians

**DOI:** 10.3390/e26100843

**Published:** 2024-10-06

**Authors:** Ali Al-Bayaty, Marek Perkowski

**Affiliations:** Department of Electrical and Computer Engineering, Portland State University, Portland, OR 97201, USA; h8mp@pdx.edu

**Keywords:** Boolean oracles, phase oracles, logical structures, logic synthesis, Hamiltonians, QAOA

## Abstract

A new methodology is introduced to solve classical Boolean problems as Hamiltonians, using the quantum approximate optimization algorithm (QAOA). This methodology is termed the “Boolean-Hamiltonians Transform for QAOA” (BHT-QAOA). Because a great deal of research and studies are mainly focused on solving combinatorial optimization problems using QAOA, the BHT-QAOA adds an additional capability to QAOA to find all optimized approximated solutions for Boolean problems, by transforming such problems from Boolean oracles (in different structures) into Phase oracles, and then into the Hamiltonians of QAOA. From such a transformation, we noticed that the total utilized numbers of qubits and quantum gates are dramatically minimized for the generated Hamiltonians of QAOA. In this article, arbitrary Boolean problems are examined by successfully solving them with our BHT-QAOA, using different structures based on various logic synthesis methods, an IBM quantum computer, and a classical optimization minimizer. Accordingly, the BHT-QAOA will provide broad opportunities to solve many classical Boolean-based problems as Hamiltonians, for the practical engineering applications of several algorithms, digital synthesizers, robotics, and machine learning, just to name a few, in the hybrid classical-quantum domain.

## 1. Introduction

The quantum approximate optimization algorithm (QAOA) was introduced by Farhi et al. [1,2] to mainly solve combinatorial optimization problems, such as the MaxCut [3,4], in the quantum domain. The MaxCut problem is a subset of the classical graph theoretical problems, which is represented by a number of nodes (*n*) connected through a set of edges {j, k}, where j and k are the indices of two connected nodes. The solutions for a MaxCut problem can be determined by finding the maximum number of cuts (in edges) for the connected nodes. In the quantum domain, the QAOA represents the MaxCut problem in the form of an ansatz Hamiltonian oracle, which is termed the “Hamiltonian clauses (*H_C_*)”, and ansatz Hamiltonian operator, which is termed the “Hamiltonian mixer (*H_M_*)”.

The wording “ansatz” means that *H_C_* and *H_M_* consist of parameterized rotational quantum gates of Pauli-Z (RZ) and Pauli-X (RX), respectively [1,2], such that *H_C_* consists of a number of RZ(*v*·*ɣ*), RZZ(*v*·*ɣ*), RZZZ(*v*·*ɣ*), and so on, while *H_M_* consists of *n* numbers of RX(*ω*·*β*), where *v* and *ω* are the coefficients (as the time evolutions for both *H_C_* and *H_M_*), *ɣ* and *β* are the parameterized angular rotations, and *n* is the number of input qubits. Note that *ɣ* and *β* are rotated between the angles of [0, 2π] and [0, π], respectively [1,2].

In general, the following steps illustrate the complete construction of QAOA, to find all approximated solutions for a classical MaxCut problem:

1. All *n* nodes of the MaxCut problem are represented into their equivalent *n* input qubits, which are initially set to the |0⟩ state.
2. Hadamard (H) gates are applied to all *n* input qubits, to create the complete quantum search space of {0,1}^⊗^*^n^* for QAOA to find all solutions.
3. Hamiltonian *H_C_* represents the quantum circuit of a MaxCut problem as the unitary operator $\left(e^{-\;i\gamma H_{C}}\right)$, which is a set of non-connected nodes as RZj(*v*·*ɣ*) and connected nodes as RZjZk(*v*·*ɣ*), where j and k are the indices of input qubits.
4. Hamiltonian *H_M_* represents the quantum circuit for the sum of all *n* input qubits as the unitary operator $\left(e^{-\;i\beta H_{M}}\right)$, which is a set of *n* RX(*ω*·*β*). Note that *H_M_* acts as the quantum diffusion operator of QAOA analogous to the diffusion operator in Grover’s algorithm [5,6,7,8], and *H_M_* may include other variants and types of gates, not just RX gates, depending on the model of QAOA used (see the Related Work section).
5. To improve the quality of all approximated solutions, *H_C_* and *H_M_* are iterated for a number of repetitions (*p*), where *p* ≥ 1, such that every $e^{-i\gamma_{p}H_{C}}$ consists of RZ(*v*·*ɣ_p_*), RZZ(*v*·*ɣ_p_*), etc., and every $e^{-\;i\beta_{p}H_{M}}$ consists of RX(*ω*·*β_p_*).
6. The numerical values of coefficients (*v* and *ω*) are calculated during the construction of *H_C_* and *H_M_* in the classical domain.
7. The numerical values of angles (*ɣ* and *β*) are initially randomized as [*ɣ*_1_…*ɣ_p_*, *β*_1_…*β_p_*] for *H_C_* and *H_M_*, respectively, in the classical domain. Note that some studies initialized such angles to defined values using machine learning and tensor techniques [9,10,11,12,13].
8. The quantum circuit of QAOA (H {*H_C_ H_M_*}*^p^*) is executed with a quantum processing unit (QPU) and then measured (in the classical domain) for approximated solutions depending on the chosen values of *ɣ* and *β*.
9. The measured solutions (as the energy cost of QAOA [1,2]), the chosen values of *ɣ* and *β* (as the optimization parameters of QAOA), and the Hamiltonians (*H_C_* and *H_M_* as an objective function) are fed to a classical optimization minimizer [14,15,16]. This minimizer re-calculates the numerical values of these optimization parameters based on the energy cost from the objective function and updates the *H_C_* and *H_M_* of QAOA with a new set of optimized numerical values of *ɣ* and *β*, respectively.
10. For a number of objective function evaluations (*nfev*), Steps 8 and 9 are concurrently repeated between a QPU and a minimizer, until finding all optimized approximated solutions for a MaxCut problem or stopping based on a pre-defined “halt condition”.

The aforementioned ten steps show why QAOA is considered a variational quantum algorithm solving combinatorial optimization problems in the hybrid classical-quantum domain [17,18,19]. The wording “variational” is equivalent to the meaning of “ansatz”.

The goal of this article is to introduce a new methodology to solve classical Boolean problems as Hamiltonians (*H_C_* and *H_M_*) using QAOA. For that, our methodology is termed the “Boolean-Hamiltonians Transform for QAOA” (BHT-QAOA), which can be summarized as follows. Firstly, an arbitrary classical Boolean problem is constructed as a quantum Boolean oracle [8,20]. This constructed oracle can be expressed in arbitrary structures, such as the Product-Of-Sums (POS) [21,22], Sum-Of-Products (SOP) [21,23], Exclusive-or Sum-Of-Products (ESOP) [24,25], XOR-Satisfiability (CNF-XOR SAT and DNF-XOR SAT) [26,27], and Algebraic Normal Form (ANF) (or Reed–Muller expansion) [28,29], just to name a few. Secondly, this constructed oracle (in any structure) is converted into its equivalent quantum Boolean oracle in ESOP structure, unless it was initially constructed in ESOP structure. Thirdly, the quantum Boolean oracle in ESOP structure is transformed into its equivalent quantum Phase oracle [8,20]. Fourthly, the Hamiltonians (*H_C_* and *H_M_*) of QAOA are generated from this transformed quantum Phase oracle, based on our modified composition rules originally presented by Hadfield [30]. Finally, all the above-mentioned ten steps of QAOA are performed in sequence using the generated *H_C_* and *H_M_*.

Please observe that a quantum Boolean oracle is easier and more straightforward in expressing an arbitrary classical Boolean problem than a quantum Phase oracle, because (i) the quantum Boolean-based gates can be directly realized using the truth tables (and De Morgan’s Laws [21]) of their equivalent classical Boolean gates, and (ii) the quantum Boolean-based gates and their qubits can be easily analyzed using classical Boolean logic, such as a Boolean logic of ‘0’ represents a quantum state of |0⟩ and a Boolean logic of ‘1’ represents a quantum state of |1⟩. For ease of description, the quantum Boolean oracle and the quantum Phase oracle will be simply denoted as the “Boolean oracle” and the “Phase oracle”, respectively.

In BHT-QAOA, converting a Boolean oracle (in any structure) into a Phase oracle will (i) remove all ancilla qubits (including the output qubit), i.e., the total number of utilized qubits will be dramatically reduced to the number of input qubits only, and (ii) omit the mirror (as the uncomputing part) of an oracle, i.e., the total number of quantum gates are significantly minimized for the quantum circuit of a Phase oracle, depending on the initial construction of a Boolean oracle that expresses a classical Boolean problem.

In this article, arbitrary classical Boolean problems (as applications) are expressed as Boolean oracles in various structures, and these Boolean oracles are then solved using BHT-QAOA for *p* repetitions, with an IBM QPU and SciPy optimization minimizer [31]. These applications are (i) an arbitrary Boolean problem in POS structure, (ii) an arbitrary Boolean problem in SOP structure, (iii) an arbitrary Boolean problem in ESOP structure, (iv) a 2 × 2 Sudoku game, and (v) a 4-bit conditioned half-adder digital circuit. Eventually, our proposed BHT-QAOA successfully finds all optimized approximated solutions for these applications, as a proof of concept for utilizing BHT-QAOA to solve arbitrary and practical classical Boolean problems in the hybrid classical-quantum domain.

## 2. Related Work

Various research works and different implementations have been proposed to focus on enhancing the optimization workflow of the following essential topics of QAOA:

1. Solving combinatorial optimization problems, such as graphs, *k*-SAT, and MaxCut problems [3,4,32,33], where *k* ≥ 3 literals (inputs).
2. Finding the optimized numerical values of the angles (*ɣ* and *β*) for the Hamiltonians (*H_C_* and *H_M_*), respectively, with fewer function evaluations (*nfev*) and repetitions (*p*) [34,35,36,37,38], or initially using machine learning techniques [9,10,11,12,13].
3. Developing variants of *H_M_* for a better QAOA in finding all optimized approximated solutions for combinatorial optimization problems [11,39,40,41,42,43,44,45].

On the other hand, Hadfield [30] proposed a set of composition rules for constructing the Hamiltonians (*H_f_*), to represent a wide variety of Boolean operators and functions. These Hamiltonians (*H_f_*) are then combined to generate simpler clauses (building blocks) of the final Hamiltonians for the applications of quantum annealing [46,47] and QAOA.

In BHT-QAOA, based on our modified sets of Hadfield’s composition rules, the Hamiltonians (*H_C_* and *H_M_*) of QAOA are simply generated from a Phase oracle, which is transformed from a Boolean oracle (in any structure) representing a Boolean problem.

## 3. Materials and Methods

**Figure 1:**
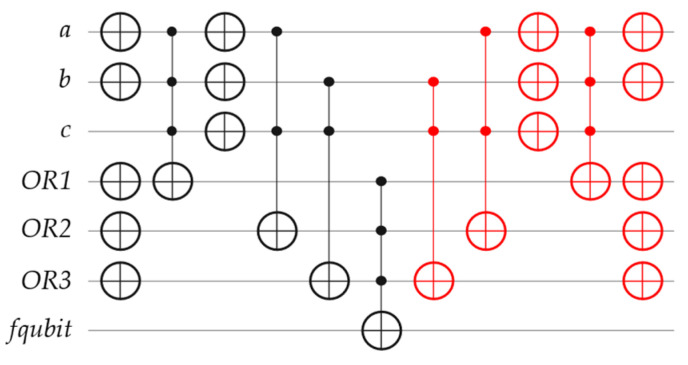
The quantum circuit of a Boolean oracle in POS structure for the classical Boolean problem (a ∨ b ∨ ¬ c) ∧ (¬ a ∨ c) ∧ (¬ b ∨ c), where the *OR1* ancilla qubit represents the term (a ∨ b ∨ ¬ c), the *OR2* ancilla qubit represents the term (¬ a ∨ c), the *OR3* ancilla qubit represents the term (¬ b ∨ c), the *fqubit* ancilla qubit performs all AND operations (∧), and the quantum gates in red denote the mirror (as the uncomputing part) of this Boolean oracle to reset all ancilla qubits to their initial quantum states. Note that all ancilla qubits are initially set to the |0⟩ states.

The essential methodology of BHT-QAOA can be discussed and evaluated using the following illustrative example. Assume an arbitrary classical Boolean problem is given, as stated in Equation (1) below, and then expressed as a Boolean oracle in POS structure, as shown in Figure 1. The following subsections discuss how to (i) convert this Boolean oracle in POS structure into the Boolean oracle in ESOP structure, (ii) transform the Boolean oracle in ESOP structure into the Phase oracle, (iii) generate the Hamiltonians (*H_C_* and *H_M_*) based on the transformed Phase oracle, and (iv) construct the overall architecture of BHT-QAOA for *H_C_* and *H_M_* in *p* repetitions, where *p* ≥ 1. Note that, for other classical Boolean problems, differently expressed Boolean oracles (in any structure) can simply follow the same steps of conversion, transformation, and generation stated in these subsections, and the *fqubit* (functional qubit) is the output ancilla qubit of a Boolean oracle (in any structure).

(*a* ∨ *b* ∨ ¬ *c*) ∧ (¬ *a* ∨ *c*) ∧ (¬ *b* ∨ *c*) (1)

### 3.1. Converting Boolean Oracles from Any Structure to ESOP Structure

There are many synthesis methods to convert a Boolean oracle (in any structure) to its equivalent Boolean oracle in ESOP structure. These synthesis methods include ESOP synthesis [24], Karnaugh map synthesis [21], and binary decision diagram (BDD) synthesis [48,49], just to name a few. For instance, the Karnaugh map synthesis for a Boolean oracle (from any structure to ESOP structure) can be summarized in the following steps.

1. Sketch an empty Karnaugh map with literals (*a*, *b*, *c*, …) and their binary Gray codes.
2. Evaluate a Boolean oracle (in any structure) for solutions (as the true minterms of ‘1’) and non-solutions (as the false minterms of ‘0’).
3. Group all solutions together from step 2, using 1-cell groups, 2-cell groups, etc.
4. Formulate each group from step 3, to generate products (∧) of literals.
5. XOR (⊕) all formulated groups together from step 4, to generate an ESOP structure.

**Figure 2:**
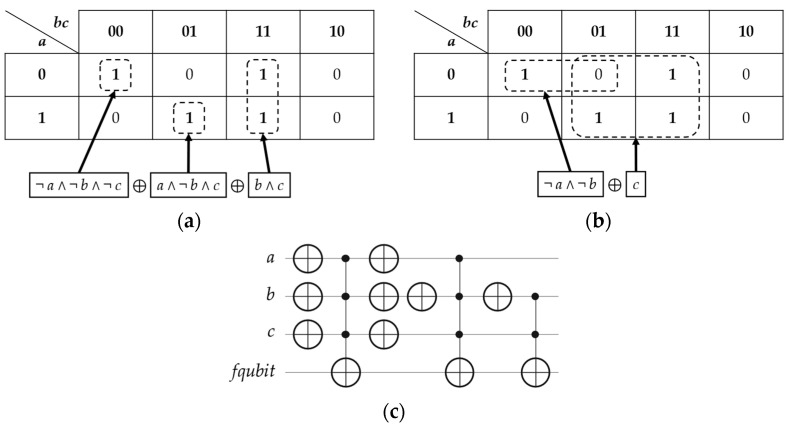
The synthesis method for the classical Boolean problem of Equation (1) above: (**a**) the Karnaugh map synthesis from the Boolean oracle (in Figure 1) to the Boolean oracle in (**c**), based on grouping all true minterms ‘1’ (as solutions) only, (**b**) the Karnaugh map synthesis from the Boolean oracle (in Figure 1) to its equivalent Boolean oracle in ESOP structure ((¬ *a* ∧ ¬ *b*) ⊕ *c*), based on grouping all ‘1’ minterms (as solutions) with one ‘0’ minterm (as an XORed solution), and (**c**) the Boolean oracle in DSOP structure of Equation (2) above, where the *fqubit* performs all XORing operations (⊕).

From Equation (1) above, since the Boolean oracle in POS structure is simple, the Karnaugh map synthesis is utilized to convert it to the Boolean oracle in ESOP structure, as stated in Equation (2) below and illustrated in Figure 2a,b, and the final quantum circuit of this Boolean oracle in ESOP structure is shown in Figure 2c. Note that in Figure 2c, (i) there is no need to optimize this Boolean oracle by removing the identical neighboring X gates, since all these gates are required for generating Hamiltonians (*H_C_* and *H_M_*) and calculating their coefficients (*v* and *ω*), respectively, and (ii) all mirrored gates and ancillae (except for *fqubit*) are removed when a Boolean oracle is in ESOP structure.

(¬ *a* ∧ ¬ *b* ∧ ¬ *c*) ⊕ (*a* ∧ ¬ *b* ∧ *c*) ⊕ (*b* ∧ *c*) (2)

Please observe that the aforementioned steps of Karnaugh map synthesis may not generate the minimized ESOP structure, as shown in Figure 2b, since these steps usually create the DSOP (Disjoint Sum-Of-Products) structure, as shown in Figure 2c, which is an expensive structure as compared to the minimized ESOP structure depending on the numbers of *n*-bit Toffoli gates, where *n* ≥ 3 qubits. However, we just want to illustrate how to convert a Boolean oracle (in any structure) to its equivalent Boolean oracle in ESOP (or DSOP) structure.

### 3.2. Transforming Boolean Oracles in ESOP Structure to Phase Oracles

**Figure 3:**
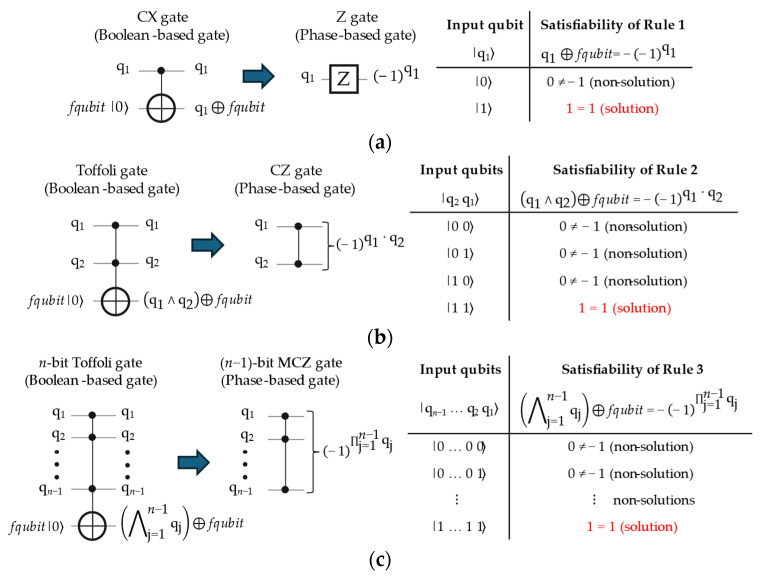
Schematics of our generalized transformation rules from the quantum Boolean-based gates (Feynman, Toffoli, and *n*-bit Toffoli gates) to the quantum Phase-based gates (Z, CZ, and (*n*−1)-bit MCZ gates) with their truth tables, where *n* ≥ 3 qubits (q inputs + *fqubit*), and texts in red indicate the solutions: (**a**) Rule 1 transforms a Feynman (CX) gate into a Z gate, (**b**) Rule 2 transforms a Toffoli gate into a CZ gate, and (**c**) Rule 3 transforms an *n*-bit Toffoli gate to an (*n*−1)-bit MCZ gate. Note that the total number of qubits is reduced by one after applying these rules, i.e., the *fqubit* is removed.

In this article, we utilize the technique originally discussed by Figgatt et al. [20] for transforming 4-bit Toffoli gates to 3-bit controlled-Z (CCZ) gates, for Grover’s algorithm of single-solution [5,6,7,8]. We efficiently generalize their technique to involve the Feynman (CX) and *n*-bit Toffoli gates, where *n* ≥ 3 qubits, for transforming Boolean oracles in ESOP structure to their equivalent Phase oracles, as presented in the following three rules and shown in Figure 3. For that, we termed these rules the “generalized transformation rules”.

**Rule 1:** A Feynman (CX) gate is transformed into a Pauli-Z (Z) gate when Equation (3) stated below is a solution-satisfiable as demonstrated in Figure 3a, where j is the index of an input qubit (q). The left side of Equation (3) is the Boolean-based output of a CX gate, and its right side is the phase-inverted output of a Z gate.

(3) $$q_{j}⊕\mathrm{fqubit}\;=-{\left(-\;1\right)}^{q_{j}}$$

**Rule 2:** A Toffoli gate is transformed into a controlled-Z (CZ) gate when Equation (4) stated below is a solution-satisfiable as shown in Figure 3b, where j and k are the indices of input qubits (q). The left side of Equation (4) is the Boolean-based output of a Toffoli gate, and its right side is the phase-inverted output of a CZ gate.

(4) $$\left(q_{j}\wedge q_{k}\right)⊕\mathrm{fqubit}=-{\left(-\;1\right)}^{q_{j}\;\cdot \;q_{k}}$$

**Rule 3:** An *n*-bit Toffoli gate is transformed into an (*n*−1)-bit multi-controlled Z (MCZ) gate when Equation (5) stated below is a solution-satisfiable as shown in Figure 3c, where j is the index of an input qubit (q) and *n* ≥ 3 qubits (q + *fqubit*). The left side of Equation (5) is the Boolean-based output of an *n*-bit Toffoli gate, and its right side is the phase-inverted output of an (*n*−1)-bit MCZ gate.

(5) $$\left({⋀}_{j=1}^{n–1}q_{j}\right)⊕\mathrm{fqubit}=-{\left(-\;1\right)}^{\prod_{j=1}^{n–1}q_{j}}$$

**Figure 4:**
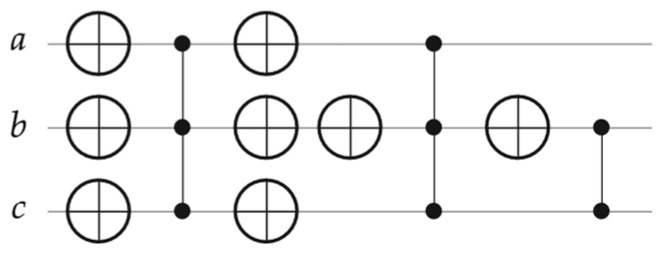
The Phase oracle for the classical Boolean problem (*a* ∨ *b* ∨ ¬ *c*) ∧ (¬ *a* ∨ *c*) ∧ (¬ *b* ∨ *c*), after applying our generalized transformation rules on its Boolean oracle in ESOP structure, such that (i) two 4-bit Toffoli gates are transformed into two 3-bit MCZ gates and one Toffoli gate into one CZ gate, (ii) all ancilla qubits (including *fqubit*) are removed, and (iii) there is no mirror for this oracle.

After applying our generalized transformation rules on the Boolean oracle in ESOP (or DSOP) structure, as stated in Equation (2) above and shown in Figure 2c, the resultant quantum circuit of the Phase oracle is then simply constructed, as illustrated in Figure 4.

Table 1 summarizes the advantages of converting the Boolean oracle in POS structure to the Boolean oracle in ESOP structure, and transforming the Boolean oracle in ESOP structure to the Phase oracle, in the context of (i) the utmost removal of all ancilla qubits (including the *fqubit*), i.e., the width of the final quantum circuit is reduced, and (ii) the dramatic minimization of multi-controlled quantum gates (after transforming the POS structure into the ESOP structure and then applying the three aforementioned generalized transformation rules), i.e., the depth of the quantum circuit is shrunk.

### 3.3. Generating Hamiltonians (H_C_ and H_M_) from Phase Oracles

Hadfield discussed, in [30], the composition rules for generating *H_C_* from a set of Hamiltonians (*H_f_*), which represent a variety of Boolean functions as simpler clauses, as stated in Table 2. In this article, to generate *H_C_* and *H_M_* from Phase oracles, we generalize some of Hadfield’s Boolean-based composition rules (*H_f_*) to Phase-based composition rules, which we termed the “generalized composition rules (*H_g_*)”. Based on our proposed three generalized transformation rules stated above, four generalized composition rules (*H_g_*) are derived from *H_f_*, as expressed in Table 3, where Rules 1, 2, and 3 of *H_g_* are simply inverting the signs (±) of their corresponding *H_f_*, for both identity (I) and RZ gates.

**Rule 1:** Performs Rule 1 of the generalized transformation rules, as stated in Equation (3) above and previously shown in Figure 3a.

**Rule 2:** Performs Rule 2 of the generalized transformation rules, as stated in Equation (4) above and previously shown in Figure 3b.

**Rule 3:** Performs Rule 3 of the generalized transformation rules, as stated in Equation (5) above and previously shown in Figure 3c.

**Rule 4:** Inverts all signs (±) of generated RZ gates in *H_g_* (Rules 1, 2, and 3), since the X gates are proposed here to only invert the phases of qubits in an *H_g_*.

**Figure 5:**
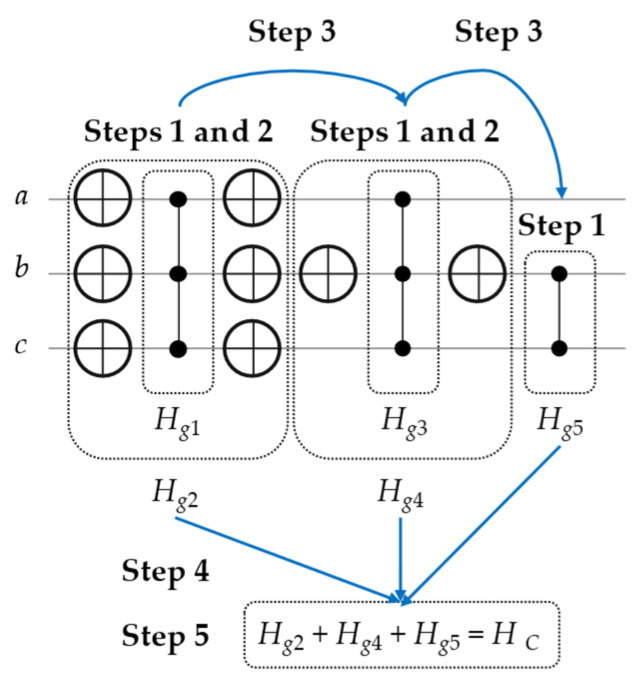
Steps of generating the Hamiltonian (*H_C_*) using our generalized composition rules (*H_g_*) from the Phase oracle shown in Figure 4, such that *H_C_* = (*H_g_*_1_ → *H_g_*_2_) + (*H_g_*_3_ → *H_g_*_4_) + *H_g_*_5_.

Consequently, from Table 3, the four generalized compositions rules (*H_g_*) will be then directly applied to the Phase oracle (shown in Figure 4) to generate *H_C_* and calculate its *v* coefficient, as demonstrated in Figure 5 and expressed in the following steps (starting from the left side of this figure).

1. Construct one *H_g_* for one Z, CZ, or MCZ, using Rule 1, Rule 2, or Rule 3, respectively.
2. If there are X gates (with their mirrored gates) surrounding Z, CZ, or MCZ in Step 1, then apply Rule 4 on *H_g_* from Step 1 to construct a new *H_g_*. If not, proceed to Step 3.
3. Repeat Steps 1 and 2 for another *H_g_* until there are no remaining Z, CZ, and MCZ.
4. Group all constructed *H_g_* into one Hamiltonian, which is *H_C_*.
5. Calculate (add or subtract) all the identical terms of *H_C_* to find the *v* coefficient.

From Table 3 and Figure 5, the Hamiltonians (*H_g_*) are step-by-step calculated, as stated in Equation (6) below. Next, the *H_C_* is simply generated from *H_g_*_2_ + *H_g_*_4_ + *H_g_*_5_, as expressed in Equation (7) below.

(6) $$\begin{array}{ll} {H_{g}}_{1} & =\frac{1}{\;8\;}\left(\left(I-Z_{a}\right)\left(-I+Z_{b}\right)\left(I-Z_{c}\right)\right)=\frac{1}{\;8\;}\left(-I+Z_{a}+Z_{b}+Z_{c}-Z_{a}Z_{b}-Z_{a}Z_{c}-Z_{b}Z_{c}+Z_{a}Z_{b}Z_{c}\right)\\ {H_{g}}_{2} & =\frac{1}{\;8\;}\left(-I-Z_{a}-Z_{b}-Z_{c}-Z_{a}Z_{b}-Z_{a}Z_{c}-Z_{b}Z_{c}-Z_{a}Z_{b}Z_{c}\right)\\ {H_{g}}_{3} & =\frac{1}{\;8\;}\left(\left(I-Z_{a}\right)\left(-I+Z_{b}\right)\left(I-Z_{c}\right)\right)=\frac{1}{\;8\;}\left(-I+Z_{a}+Z_{b}+Z_{c}-Z_{a}Z_{b}-Z_{a}Z_{c}-Z_{b}Z_{c}+Z_{a}Z_{b}Z_{c}\right)\\ {H_{g}}_{4} & =\frac{1}{\;8\;}\left(-I+Z_{a}-Z_{b}+Z_{c}+Z_{a}Z_{b}-Z_{a}Z_{c}+Z_{b}Z_{c}-Z_{a}Z_{b}Z_{c}\right)\\ {H_{g}}_{5} & =\frac{1}{\;4\;}\left(\left(I-Z_{b}\right)\left(-I+Z_{c}\right)\right)=\frac{1}{\;4\;}\left(-I+Z_{b}+Z_{c}-Z_{b}Z_{c}\right) \end{array}$$

(7) $$H_{C}={H_{g}}_{2}+{H_{g}}_{4}+{H_{g}}_{5}=-\frac{1}{2}I+\frac{1}{4}Z_{c}-\frac{1}{4}\left(Z_{a}Z_{c}+Z_{b}Z_{c}\right)-\frac{1}{4}{\;Z}_{a}Z_{b}Z_{c}$$

Hence, from Equation (7) above, *v* = [*v*_1_, *v*_2_, *v*_3_, *v*_4_] = [$-\frac{1}{\;2\;}$, $\frac{1}{\;4\;}$, $-\frac{1}{\;4\;}$, $-\frac{1}{\;4\;}$], where *v*_1_ is for all non-connected input qubits (as the identity ‘I’), *v*_2_ is for *c* input qubit only, i.e., RZ*_c_*(0.25·*ɣ*), *v*_3_ is for only two connected input qubits {*a* and *c*; *b* and *c*}, i.e., RZ*_a_*Z*_c_*(−0.25·*ɣ*) and RZ*_b_*Z*_c_*(−0.25·*ɣ*), and *v*_4_ is for all connected input qubits {*a*, *b*, *c*}, i.e., RZ*_a_*Z*_b_*Z*_c_*(−0.25·*ɣ*). For a practical quantum implementation of QAOA as well as our introduced BHT-QAOA, *H_C_* in Equation (7) is rewritten as the following format for the sequence of three qubits {*c*, *b*, *a*}, as stated in Equation (8) below, where ‘ZII’ is equivalent to RZ*_c_*, ‘ZIZ’ is equivalent to RZ*_a_*Z*_c_*, ‘ZZZ’ is equivalent to RZ*_a_*Z*_b_*Z*_c_*, and so on.

*H_C_* = ([‘III’, ‘ZII’, ‘ZIZ’, ‘ZZI’, ‘ZZZ’], *coeffs* = [−0.5, 0.25, −0.25, −0.25, −0.25]) (8)

Because *β* (as a set of rotational angles of *H_M_*) rotates between [0, π] [1,2], we set its coefficient (*ω*) to cover the entire range between [0, 2π] for possible phase values of RX gates in *H_M_*, to find all optimized approximated solutions for an arbitrary classical Boolean problem. In other words, *ω* (as the coefficient of *β*) is initially set to ‘2.0’ for all *n* numbers of RX(*ω*·*β*) gates in *H_M_*, where *n* is the total number of input qubits. Similar to the practical implementation of *H_C_*, *H_M_* is rewritten as the following format for the sequence of three qubits {*c*, *b*, *a*}, as stated in Equation (9) below, where ‘XII’ is equivalent to RX*_c_*, ‘IXI’ is equivalent to RX*_b_*, and ‘IIX’ is equivalent to RX*_a_*.

*H_M_* = ([‘XII’, ‘IXI’, ‘IIX’], *coeffs* = [2.0, 2.0, 2.0]) (9)

**Figure 6:**
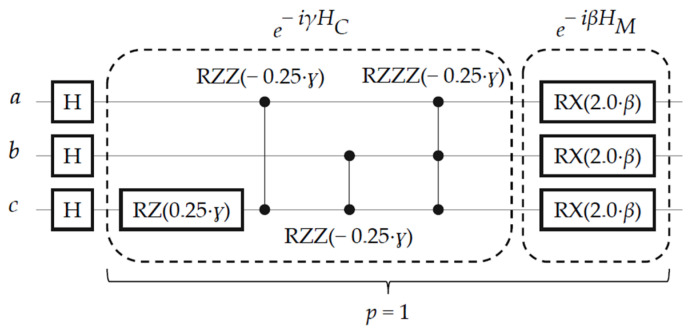
The quantum circuit for the classical Boolean problem of (*a* ∨ *b* ∨ ¬ *c*) ∧ (¬ *a* ∨ *c*) ∧ (¬ *b* ∨ *c*) after applying our generalized transformation rules (*H_g_*) to generate two Hamiltonians (*H_C_* and *H_M_*) in one repetition (*p*), where H is the Hadamard gate, *H_C_* is (‘III’, ‘ZII’, ‘ZIZ’, ‘ZZI’, ‘ZZZ’) with its coefficient *v* = [$-\frac{1}{\;2\;}$, $\frac{1}{\;4\;}$, $-\frac{1}{\;4\;}$, $-\frac{1}{\;4\;}$], *H_M_* is (‘XII’, ‘IXI’, ‘IIX’) with its coefficient *ω* = 2.0, and *ɣ* and *β* are the optimization angular parameters for *H_C_* and *H_M_*, respectively.

From Equations (8) and (9) above, the quantum circuit of BHT-QAOA for the generated Hamiltonians (*H_C_* and *H_M_*) of the Boolean problem (*a* ∨ *b* ∨ ¬ *c*) ∧ (¬ *a* ∨ *c*) ∧ (¬ *b* ∨ *c*) is eventually constructed for one repetition (*p* = 1), as illustrated in Figure 6.

### 3.4. Architecture of BHT-QAOA

**Figure 7:**
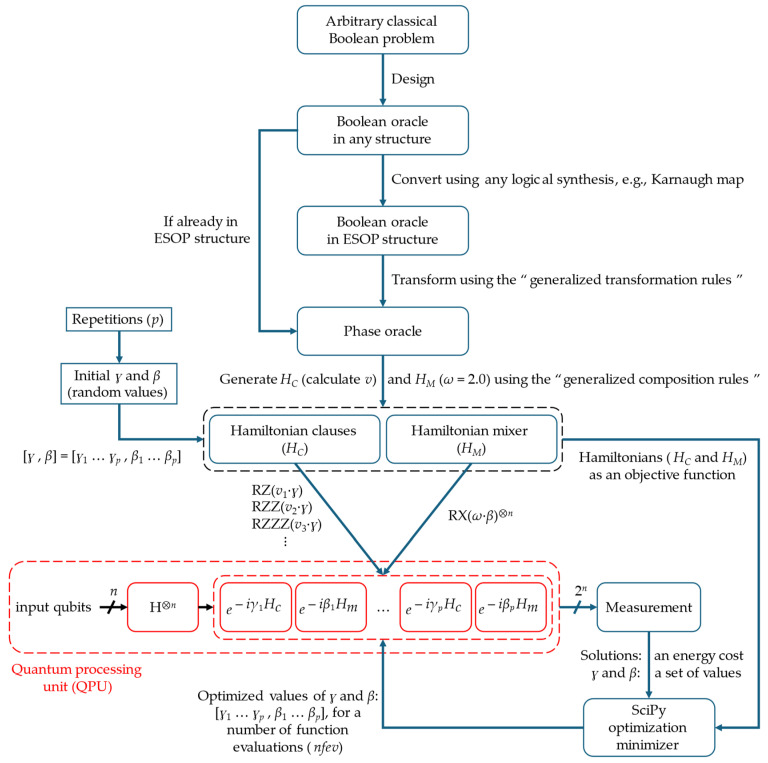
The architecture of our Boolean–Hamiltonian Transform for QAOA (BHT-QAOA) to solve arbitrary classical Boolean problems as Hamiltonians (*H_C_* and *H_M_*). The BHT-QAOA is mainly grouped into two processing domains: (i) the classical processing domain as denoted by blue, and (ii) the quantum processing domain as denoted by red.

After transforming an arbitrary classical Boolean problem to the Hamiltonians (*H_C_* and *H_M_*) and calculating their coefficients (*v* and *ω*), respectively, the numerical values of *ɣ* and *β* are initially randomized and then plugged into the architecture of BHT-QAOA for first execution, as shown in Figure 7. Subsequently, the SciPy optimization minimizer [31] is utilized to optimize these numerical values (*ɣ* and *β*) for better-approximated solutions, in a number of function evaluations (*nfev*), by employing three cofactors as follows.

1. *H_C_* and *H_M_* (in a number of *p*), as the “objective function” needs to be minimized.
2. Measured solutions of BHT-QAOA, as the “energy cost” of the objective function.
3. Previously calculated *ɣ* and *β*, as their “numerical values” need to be optimized.

For the SciPy optimization minimizer, we use the constrained optimization by linear approximation (COBYLA) algorithm [14,16,31], which is a parametric iterative method for derivative-free constrained optimization that updates and minimizes the approximation values (as *ɣ* and *β*) for an objective function (as *H_C_* and *H_M_* of lower energy cost). On the other hand, our future work will focus on finding a cost-effective minimizer algorithm, to efficiently implement BHT-QAOA with a smaller value of *p* (in the quantum domain) and fewer *nfev* (in the classical domain).

## 4. Results and Discussion

Arbitrary classical Boolean problems (applications) are designed as Boolean oracles (in different structures), and these Boolean oracles are then solved using our introduced BHT-QAOA for *p* repetitions, where *p* ≥ 1. In our experiments, the ibm_brisbane [50] QPU of 127 qubits performs the quantum processing domain of BHT-QAOA, i.e., executes the quantum circuit of an application in *p* repetitions, and the SciPy minimizer function performs the classical processing domain of BHT-QAOA, i.e., optimizes the numerical values of *ɣ* and *β* based on the minimized energy cost of their Hamiltonians (*H_C_* and *H_M_*), for a number of function evaluations (*nfev*).

Because of our IBM Quantum Platform account limitations, the complete architecture of BHT-QAOA (shown in Figure 7) is completely simulated in the classical domain using IBM quantum libraries (Qiskit, AerSimulator, and Aer-EstimatorV2 [50,51,52]), for 1024 resampling times, which are the so-called “shots” [53]. After this classically simulated BHT-QAOA, the final optimized numerical values of *ɣ* and *β* (from the SciPy minimizer) are plugged into their respective Hamiltonians (*H_C_* and *H_M_*) of the quantum circuit for an application, in which it is then executed once with ibm_brisbane QPU.

**Figure 8:**
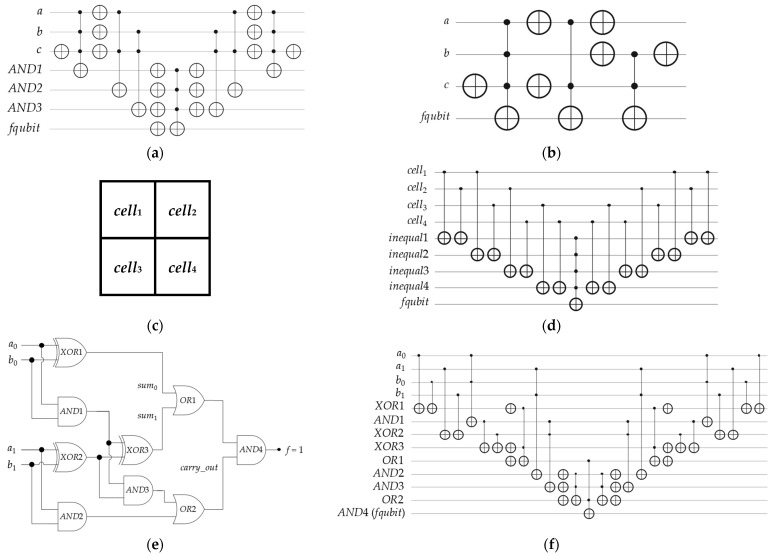
Schematics of the classical representations and the quantum circuits for arbitrary Boolean problems: ((**a**), *upper-left*) the Boolean oracle in SOP structure representing Equation (10) above, ((**b**), *upper-right*) the Boolean oracle in ESOP structure representing Equation (11) above, ((**c**), *middle-left*) the board layout of 2 × 2 Sudoku, ((**d**), *middle-right*) the Boolean oracle in CNF-XOR SAT structure of 2 × 2 Sudoku, ((**e**), *bottom-left*) the classical 4-bit conditioned half-adder for two 2-bit numbers (A = *a*_1_*a*_0_ and B = *b*_1_*b*_0_), and ((**f**), *bottom-right*) the Boolean oracle in a mixed structure representing Equation (13) above.

Please observe that due to the limited physical connectivity of four neighboring qubits for the recent quantum layouts of IBM QPUs, the constructed Boolean oracles (in various structures) for the following applications must have *n* input qubits and *m* ancilla qubits (including *fqubit*), where 2 ≤ *n* ≤ 4 and *m* ≥ 1. The *m* ancilla qubits (i) do not affect the fidelity of the final optimized approximated solutions of applications utilizing the BHT-QAOA and (ii) have no relation to the limited physical connectivity of any IBM QPU, because all these constructed Boolean oracles are transformed into Phase oracles and then into Hamiltonians (*H_C_* and *H_M_*). In other words, Hamiltonians do not have any ancilla qubits in their quantum circuits, and all *m* ancilla qubits are removed. Figure 8 demonstrates the classical representations and the quantum circuits of Boolean oracles for these applications, as follows.

1. An arbitrary Boolean problem in POS structure, as stated in Equation (1) above and shown in Figure 1.
2. An arbitrary Boolean problem in SOP structure, as stated in Equation (10) below and shown in Figure 8a.

(*a* ∧ *b* ∧ ¬ *c*) ∨ (¬ *a* ∧ *c*) ∨ (¬ *b* ∧ *c*) (10)

3. An arbitrary Boolean problem in ESOP structure, as stated in Equation (11) below and shown in Figure 8b.

(*a* ∧ *b* ∧ ¬ *c*) ⊕ (¬ *a* ∧ *c*) ⊕ (¬ *b* ∧ *c*) (11)

4. A 2 × 2 Sudoku game, which is the constraints satisfaction problem—satisfiability (CSP-SAT) [54,55], as stated in Equation (12) below and illustrated in Figure 8c,d.

(*cell*_1_ ⊕ *cell*_2_) ∧ (*cell*_1_ ⊕ *cell*_3_) ∧ (*cell*_2_ ⊕ *cell*_4_) ∧ (*cell*_3_ ⊕ *cell*_4_) (12)

5. A 4-bit conditioned half-adder digital circuit, which is ORing two 1-bit sums and then ANDing them with one 1-bit carry-out, as stated in Equation (13) below and demonstrated in Figure 8e,f.

[(*a*_0_ ⊕ *b*_0_) ∨ ((*a*_0_ ∧ *b*_0_) ⊕ (*a*_1_ ⊕ *b*_1_))] ∧ [(*a*_1_ ∧ *b*_1_) ∨ ((*a*_0_ ∧ *b*_0_) ∧ (*a*_1_ ⊕ *b*_1_))] (13)

Table 4 states the removal of all ancilla qubits (including *fqubit*) and the reduction number of quantum gates, after applying the generalized transformation rules to form Phase oracles. These Phase oracles are then transformed into the Hamiltonians (*H_C_* and *H_M_*) of BHT-QAOA. Note that, in Table 4, (i) a Pauli-X (X) gate in a Boolean oracle remains as-is for a Phase oracle, (ii) a Feynman (CX) gate in a Boolean oracle is equivalent to the controlled Pauli-Z (CZ) gate in a Phase oracle, and (iii) an *n*-bit Toffoli gate in a Boolean oracle is equivalent to an *n*-bit MCZ gate in a Phase oracle, where *n* ≥ 3 qubits.

On the one hand, the classically simulated BHT-QAOA successfully optimizes the numerical values of *ɣ* and *β* and finds all approximated solutions for these applications, as a proof of concept for utilizing our introduced BHT-QAOA in solving arbitrary classical Boolean problems in the simulated classical-quantum domain.

On the other hand, the quantum circuit of every application (using the simulated optimized numerical values of *ɣ* and *β*) is executed once with ibm_brisbane QPU for solution fidelity, as a proof of concept for utilizing BHT-QAOA to solve arbitrary classical Boolean problems in the hybrid classical-quantum domain.

**Figure 9:**
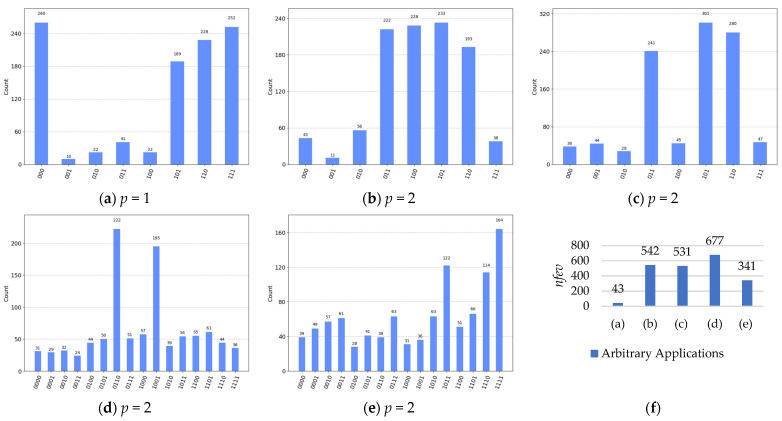
The final measured solutions for arbitrary applications executed with ibm_brisbane QPU (for 1024 shots): ((**a**), *upper-left*) the four solutions {$\overset{-}{a}\overset{-}{b}\overset{-}{c}$, $a\overset{-}{b}c$, $\overset{-}{a}\mathrm{bc}$, abc} for the Boolean oracle in POS structure of Equation (1) above, ((**b**), *upper-middle*) the four solutions {$\mathrm{ab}\overset{-}{c}$, $\overset{-}{a}\overset{-}{b}c$, $a\overset{-}{b}c$, $\overset{-}{a}\mathrm{bc}$} for the Boolean oracle in SOP structure of Equation (10) above, ((**c**), *upper-right*) the three solutions {$\mathrm{ab}\overset{-}{c}$, $a\overset{-}{b}c$, $\overset{-}{a}\mathrm{bc}$} for the Boolean oracle in ESOP structure of Equation (11) above, ((**d**), *bottom-left*) the two permutative solutions {solution 1: *cell*_1_ = *cell*_4_ = 0 and *cell*_2_ = *cell*_3_ = 1; solution 2: *cell*_1_ = *cell*_4_ = 1 and *cell*_2_ = *cell*_3_ = 0} for the 2 × 2 Sudoku game of Equation (12) above, ((**e**), *bottom-middle*) the three solutions as two 2-bit numbers {$a_{0}a_{1}\bar{b_{0}}b_{1}$, $\bar{a_{0}}a_{1}b_{0}b_{1}$, $a_{0}a_{1}b_{0}b_{1}$} for the 4-bit conditioned half-adder of Equation (13) above, and ((**f**), *bottom-right*) the required *nfev* for the SciPy optimization minimizer to successfully optimize the numerical values of *ɣ* and *β* for the above-mentioned applications, in the subfigures (**a**–**e**).

Figure 9 depicts the final measured solutions for every application from the real quantum executions using ibm_brisbane QPU. In Figure 9, the first measured bit (the upper-right) of a solution is equivalent to the first input qubit, and the last measured bit (the bottom-left) of a solution is equivalent to the last input qubit for the Boolean oracle of an arbitrary application.

As illustrated in Figure 9a–e, the histograms represent the final measured outputs for the arbitrary Boolean problems (as applications), and each histogram of an application denotes the results of (a) four solutions {$\overset{-}{a}\overset{-}{b}\overset{-}{c}$, $a\overset{-}{b}c$, $\overset{-}{a}\mathrm{bc}$, abc} for the Boolean oracle in POS structure of Equation (1) above, (b) four solutions {$\mathrm{ab}\overset{-}{c}$, $\overset{-}{a}\overset{-}{b}c$, $a\overset{-}{b}c$, $\overset{-}{a}\mathrm{bc}$} for the Boolean oracle in SOP structure of Equation (10) above, (c) three solutions {$\mathrm{ab}\overset{-}{c}$, $a\overset{-}{b}c$, $\overset{-}{a}\mathrm{bc}$} for the Boolean oracle in ESOP structure of Equation (11) above, (d) two permutative solutions {solution 1: *cell*_1_ = *cell*_4_ = 0 and *cell*_2_ = *cell*_3_ = 1; solution 2: *cell*_1_ = *cell*_4_ = 1 and *cell*_2_ = *cell*_3_ = 0} for the 2 × 2 Sudoku of Equation (12) above, and (e) three solutions {$a_{0}a_{1}\bar{b_{0}}b_{1}$, $\bar{a_{0}}a_{1}b_{0}b_{1}$, $a_{0}a_{1}b_{0}b_{1}$} for the 4-bit conditioned half-adder of Equation (13) above.

Note that, in Figure 9, (i) the first measured bit (the upper-right in a histogram) is equivalent to the first input qubit of QAOA, (ii) the final measured bit (the bottom-left in a histogram) is equivalent to the final input qubit of QAOA, (iii) *p* states the number of repetitions for the Hamiltonians (*H_C_* and *H_M_*) in the quantum circuit of QAOA, (iv) *nfev* is the number of function evaluations from the SciPy optimization minimizer, (v) ‘Count’ is the total number of 1024 shots for all input qubits, where the higher Count indicates a solution (as a higher probability of qubits measurement) and the lower Count indicates a non-solution (as a lower probability of qubits measurement), and (vi) $\overset{-}{x}$ means *x* has a false binary value of ‘0’; otherwise, *x* has a true binary value of ‘1’, where *x* is a literal (variable) in an equation that represents a Boolean problem (application), in the classical domain.

Please observe that the required *nfev* for the SciPy optimization minimizer varies and fluctuates, since such a minimizer mainly depends on two factors, as follows.

1. The initially randomized numerical values of *ɣ* and *β* for *H_C_* and *H_M_*, respectively.
2. The noisy simulation models (AerSimulator and Aer-EstimatorV2), which are utilized for simulating BHT-QAOA, in the classical domain.

Accordingly, the BHT-QAOA will provide broad opportunities to find all solutions for many classical Boolean problems constructed as Hamiltonians (*H_C_* and *H_M_*), which are neither designed nor solved using the standard QAOA [1,2]. Therefore, various classical Boolean problems for the applications of digital logic circuits, synthesizers, robotics, and machine learning can be realized as Hamiltonians and then solved using BHT-QAOA in the hybrid classical-quantum domain.

## 5. Conclusions

A new methodology is introduced to solve arbitrary classical Boolean problems as Hamiltonians (*H_C_* and *H_M_*), using the quantum approximate optimization algorithm (QAOA) [1,2]. Our methodology is termed the “Boolean-Hamiltonians Transform for QAOA” (BHT-QAOA), which is summarized as follows: (i) an arbitrary classical Boolean problem is expressed as a Boolean oracle in arbitrary structures, e.g., POS, SOP, ESOP, and XOR SAT, just to name a few, (ii) this Boolean oracle (in any structure) is converted into its equivalent Boolean oracle in ESOP structure, unless it was firstly constructed in ESOP structure, (iii) this Boolean oracle in ESOP structure is transformed into its equivalent Phase oracle based on our modified transformations of Toffoli gates, which are originally presented by Figgatt et al. [20], (iv) the Hamiltonians (*H_C_* and *H_M_*) are generated from this transformed Phase oracle based on our modified set of Hamiltonian compositions, which are originally presented by Hadfield [30], and (v) all execution steps of the standard QAOA are performed on the generated *H_C_* and *H_M_*. A classical optimization minimizer is utilized to find better-approximated solutions for an arbitrary classical Boolean problem based on the optimized numerical values of *ɣ* and *β* angles for *H_C_* and *H_M_*, respectively.

In BHT-QAOA, all ancilla qubits (including the output qubit) and the mirror (as the uncomputing part) of a quantum circuit will be completely removed when transforming a Boolean oracle (in any structure) into its equivalent Phase oracle. In other words, during the conversion and transformation steps of BHT-QAOA, the total number of utilized qubits will be dramatically reduced to the number of input qubits only, and the total number of quantum gates will be significantly minimized for the final quantum circuit of a Phase oracle, for an arbitrary classical Boolean problem.

In this article, arbitrary Boolean applications are constructed as Boolean oracles (in various structures), and then BHT-QAOA successfully finds all optimized approximated solutions for these applications using a classical optimization minimizer and an IBM quantum computer, since our introduced BHT-QAOA is considered as a hybrid classical-quantum algorithm. In conclusion, further classical Boolean problems can be constructed as Boolean oracles (in mixed structures) for the practical engineering applications in the topics of digital synthesizers, computer vision, robotics, and machine learning, just name a few, and BHT-QAOA will successfully solve such practical applications effectively in the hybrid classical-quantum domain.

## Figures and Tables

**Table 1:** Comparison of the number of qubits and quantum gates for the Boolean and Phase oracles for the Boolean problem of Equation (1) stated above, after applying the generalized transformation rules.

Oracular Problems	Number of Qubits			Number of Multi-Controlled Gates			Quantum Circuit Required a Mirror?
	Inputs	Ancillae (with *fqubit*)	Total	Feynman (CX)	3-bit Toffoli	4-bit Toffoli	
Boolean oracle in POS structure	3	4	7	0	4	3	Yes
Boolean oracle in ESOP structure	3	1	4	0	1	2	No
Phase oracle	3	0	3	1 (as a CZ)	2 (as a CCZ)	0	No

**Table 2:** Some of Hadfield’s Boolean-based composition rules (*H_f_*) [30], where j and k are the indices of input qubits (q), Z_j_ is the RZ gate applied on q_j_, and *fqubit* initially sets to the |0⟩ state.

Gate	Type	*f*(*x*)	*H_f_*
Feynman (CX)	Boolean	q_j_ ⊕ *fqubit* = q_j_	$\frac{1}{\;2\;}\;I\;-\frac{1}{\;2\;}Z_{j}$
Toffoli	Boolean	q_j_ ∧ q_k_	$\frac{1}{\;4\;}\;I\;-\frac{1}{\;4\;}\left(Z_{j}+Z_{k}-Z_{j}Z_{k}\right)$
*n*-bit Toffoli	Boolean	${⋀}_{\;j=1}^{n-1}q_{j}$	$\frac{1}{{\;2}^{n–1}}\prod_{\;j=1}^{n–1}\left(\;I\;-Z_{j}\right)$

**Table 3:** Our proposed generalized composition rules (*H_g_*) for Phase oracles, where j and k are the indices of input qubits (q), Z_j_ is the RZ gate applied on q_j_, Q = {q_j_, q_k_…q_j_q_k_…}, and Z_Q_ = {Z_j_, Z_k_…Z_j_Z_k_…}.

Rules	Gate	Type	*g*(*x*)	*H_g_*
**Rule 1**	Pauli-Z (Z)	Phase	${\left(-1\right)}^{q_{j}}$	$-\frac{1}{\;2\;}\;I+\frac{1}{\;2\;}Z_{j}$
**Rule 2**	CZ	Phase	${\left(-1\right)}^{q_{j}\cdot q_{k}}$	$-\frac{1}{\;4\;}\;I+\frac{1}{\;4\;}\left(Z_{j}+Z_{k}-Z_{j}Z_{k}\right)$
**Rule 3**	MCZ	Phase	${\left(-1\right)}^{\prod_{j=1}^{n}q_{j}}$	$\frac{1}{{\;2}^{n}}\prod_{\;j=1}^{n}{\left(-\;1\right)}^{\;j+1}\left(\;I-Z_{j}\right)$
**Rule 4**	Pauli-X (X)	Phase	${\left(-1\right)}^{\forall j\;\in \;Q}$	Invert signs (±) of all jth qubits in Z_Q_

**Table 4:** The effect of generalized transformation rules on removing all ancilla qubits (including *fqubit*) and reducing the numbers of quantum gates for the final Phase oracles for BHT-QAOA.

	Entities in a Boolean Oracle → Entities in a Phase Oracle				
Qubits and Quantum Gates (Entities)	Arbitrary Problem in POS	Arbitrary Problem in SOP	Arbitrary Problem in ESOP	2 × 2 Sudoku Game	4-bit Conditioned Half-Adder Circuit
Input qubits	3 → 3	3 → 3	3 → 3	4 → 4	4 → 4
Ancilla qubits	4 → 0	4 → 0	1 → 0	5 → 0	9 → 0
Pauli-X (X)	16 → 8	15 → 6	6 → 6	0 → 8	12 → 2
Feynman (CX)	0 → 1	0 → 1	0 → 2	16 → 0	12 → 0
3-bit Toffoli	4 → 2	4 → 2	2 → 1	–	11 → 1
4-bit Toffoli	3 → 0	3 → 0	1 → 0	0 → 2	0 → 1
5-bit Toffoli	–	–	–	1 → 0	–

## Data Availability

The original contributions presented in our study are included in this article; further inquiries can be directed to the corresponding author (A.A.-B.).

## References

[1] Farhi E., Goldstone J., Gutmann S. (2014). A quantum approximate optimization algorithm. arXiv.

[2] Farhi E., Goldstone J., Gutmann S., Neven H. (2017). Quantum algorithms for fixed qubit architectures. arXiv.

[3] Goemans M.X., Williamson D.P. 878-approximation algorithms for max cut and max 2sat. Proceedings of the Twenty-Sixth Annual ACM Symposium on the Theory of Computing.

[4] Rendl F., Rinaldi G., Wiegele A. (2010). Solving max-cut to optimality by intersecting semidefinite and polyhedral relaxations. Math. Program..

[5] Grover L.K. (1996). A fast quantum mechanical algorithm for database search. Proceedings, 28th Annual ACM Symposium on the Theory of Computing.

[6] Grover L.K. (1997). Quantum mechanics helps in searching for a needle in a haystack. Phys. Rev. Lett..

[7] Grover L.K. (1998). A framework for fast quantum mechanical algorithms. Proceedings of the Thirtieth Annual ACM Symposium on Theory of Computing.

[8] Al-Bayaty A., Perkowski M. (2023). A concept of controlling Grover diffusion operator: A new approach to solve arbitrary Boolean-based problems. Res. Sq..

[9] Moussa C., Wang H., Bäck T., Dunjko V. (2022). Unsupervised strategies for identifying optimal parameters in quantum approximate optimization algorithm. EPJ Quantum Technol..

[10] Amosy O., Danzig T., Lev O., Porat E., Chechik G., Makmal A. (2024). Iteration-free quantum approximate optimization algorithm using neural networks. Quantum Mach. Intell..

[11] Herrman R., Lotshaw P.C., Ostrowski J., Humble T.S., Siopsis G. (2022). Multi-angle quantum approximate optimization algorithm. Sci. Rep..

[12] Wurtz J., Lykov D. (2021). Fixed-angle conjectures for the quantum approximate optimization algorithm on regular MaxCut graphs. Phys. Rev. A.

[13] Crooks G.E. (2018). Performance of the quantum approximate optimization algorithm on the maximum cut problem. arXiv.

[14] Fernández-Pendás M., Combarro E.F., Vallecorsa S., Ranilla J., Rúa I.F. (2022). A study of the performance of classical minimizers in the quantum approximate optimization algorithm. J. Comput. Appl. Math..

[15] Powell M.J.D., Gomez S., Gomez S., Hennart J.P. (1994). Advances in Optimization and Numerical Analysis.

[16] Powell M.J.D. (2007). A view of algorithms for optimization without derivatives. Math. Today-Bull. Inst. Math. Its Appl..

[17] Cerezo M., Arrasmith A., Babbush R., Benjamin S.C., Endo S., Fujii K., McClean J.R., Mitarai K., Yuan X., Cincio L. (2021). Variational quantum algorithms. Nat. Rev. Phys..

[18] Wecker D., Hastings M.B., Troyer M. (2015). Progress towards practical quantum variational algorithms. Phys. Rev. A.

[19] Tilly J., Chen H., Cao S., Picozzi D., Setia K., Li Y., Grant E., Wossnig L., Rungger I., Booth G.H. (2022). The variational quantum eigensolver: A review of methods and best practices. Phys. Rep..

[20] Figgatt C., Maslov D., Landsman K.A., Linke N.M., Debnath S., Monroe C. (2017). Complete 3-qubit Grover search on a programmable quantum computer. Nat. Commun..

[21] Wakerly J.F. (2014). Digital Design: Principles and Practices.

[22] Zhang L.X., Huang W. (2007). A note on the invariance principle of the product of sums of random variables. Electron. Commun. Probab..

[23] Zimmermann R., Tran D.Q. (2003). Optimized synthesis of sum-of-products. Thrity-Seventh Asilomar Conference on Signals, Systems & Computers.

[24] Mishchenko A., Perkowski M. (2001). Fast heuristic minimization of exclusive sums-of-products. 5th Int. Workshop on Applications of the Reed-Muller Expansion in Circuit Design.

[25] Sasao T. (1993). EXMIN2: A simplification algorithm for exclusive-or-sum-of-products expressions for multiple-valued-input two-valued-output functions. IEEE Trans. Comput.-Aided Des. Integr. Circuits Syst..

[26] Ibrahimi M., Kanoria Y., Kraning M., Montanari A. (2012). The set of solutions of random XORSAT formulae. Proceedings of the Twenty-Third Annual ACM-SIAM Symposium on Discrete Algorithms.

[27] Soos M., Meel K.S. (2019). BIRD: Engineering an efficient CNF-XOR SAT solver and its applications to approximate model counting. Proc. AAAI Conf. Artif. Intell..

[28] Stankovic R.S., Sasao T. (2001). A discussion on the history of research in arithmetic and Reed-Muller expressions. IEEE Trans. Comput.-Aided Des. Integr. Circuits Syst..

[29] Kurgalin S., Borzunov S. (2021). Concise Guide to Quantum Computing: Algorithms, Exercises, and Implementations.

[30] Hadfield S. (2021). On the representation of Boolean and real functions as Hamiltonians for quantum computing. ACM Trans. on Quantum Comput. (TQC).

[31] Lavrijsen W., Tudor A., Müller J., Iancu C., De Jong W. (2020). Classical optimizers for noisy intermediate-scale quantum devices. 2020 IEEE International Conference on Quantum Computing and Engineering (QCE).

[32] Boulebnane S., Montanaro A. (2022). Solving Boolean satisfiability problems with the quantum approximate optimization algorithm. arXiv.

[33] Akshay V., Philathong H., Morales M.E., Biamonte J.D. (2020). Reachability deficits in quantum approximate optimization. Phys. Rev. Lett..

[34] Mandl A., Barzen J., Bechtold M., Leymann F., Wild K. (2024). Amplitude amplification-inspired QAOA: Improving the success probability for solving 3sat. Quantum Sci. Technol..

[35] Lin C.Y.Y., Zhu Y. (2016). Performance of QAOA on typical instances of constraint satisfaction problems with bounded degree. arXiv.

[36] Bärtschi A., Eidenbenz S. (2020). Grover mixers for QAOA: Shifting complexity from mixer design to state preparation. 2020 IEEE International Conference on Quantum Computing and Engineering (QCE).

[37] Zhang Z., Paredes R., Sundar B., Quiroga D., Kyrillidis A., Duenas-Osorio L., Pagano G., Hazzard K.R. (2024). Grover-QAOA for 3-sat: Quadratic speedup, fair-sampling, and parameter clustering. arXiv.

[38] Weidenfeller J., Valor L.C., Gacon J., Tornow C., Bello L., Woerner S., Egger D.J. (2022). Scaling of the quantum approximate optimization algorithm on superconducting qubit based hardware. Quantum.

[39] Blekos K., Brand D., Ceschini A., Chou C.H., Li R.H., Pandya K., Summer A. (2024). A review on quantum approximate optimization algorithm and its variants. Phys. Rep..

[40] Govia L.C.G., Poole C., Saffman M., Krovi H.K. (2021). Freedom of the mixer rotation axis improves performance in the quantum approximate optimization algorithm. Phys. Rev. A.

[41] Bravyi S., Kliesch A., Koenig R., Tang E. (2020). Obstacles to variational quantum optimization from symmetry protection. Phys. Rev. Lett..

[42] Golden J., Bärtschi A., O’Malley D., Eidenbenz S. (2021). Threshold-based quantum optimization. 2021 IEEE International Conference on Quantum Computing and Engineering (QCE).

[43] Wang Z., Rubin N.C., Dominy J.M., Rieffel E.G. (2020). XY mixers: Analytical and numerical results for the quantum alternating operator ansatz. Phys. Rev. A.

[44] Vijendran V., Das A., Koh D.E., Assad S.M., Lam P.K. (2024). An expressive ansatz for low-depth quantum approximate optimisation. Quantum Sci. Technol..

[45] Sarmina B.G., Sun G.H., Dong S.H. (2023). Principal component analysis and t-distributed stochastic neighbor embedding analysis in the study of quantum approximate optimization algorithm entangled and non-entangled mixing operators. Entropy.

[46] Yarkoni S., Raponi E., Bäck T., Schmitt S. (2022). Quantum annealing for industry applications: Introduction and review. Rep. Prog. Phys..

[47] Morita S., Nishimori H. (2008). Mathematical foundation of quantum annealing. J. Math. Phys..

[48] Ebendt R., Fey G., Drechsler R. (2005). Advanced BDD Optimization.

[49] Wille R., Drechsler R. (2010). Effect of BDD optimization on synthesis of reversible and quantum logic. Electron. Notes Theor. Comput. Sci..

[50] Karimi N., Elyasi S.N., Yahyavi M. (2024). Implementation and measurement of quantum entanglement using IBM quantum platforms. Phys. Scr..

[51] Wille R., Van Meter R., Naveh Y. (2019). IBM’s Qiskit tool chain: Working with and developing for real quantum computers. 2019 Design, Automation & Test in Europe Conf. & Exhibition (DATE).

[52] Georgopoulos K., Emary C., Zuliani P. (2021). Modeling and simulating the noisy behavior of near-term quantum computers. Phys. Rev. A.

[53] Rao P., Yu K., Lim H., Jin D., Choi D. (2020). Quantum amplitude estimation algorithms on IBM quantum devices. Quantum Communications and Quantum Imaging XVIII.

[54] Simonis H. (2005). Sudoku as a constraint problem. CP Workshop on Modeling and Reformulating Constraint Satisfaction Problems.

[55] Lynce I., Ouaknine J. Sudoku as a SAT problem. Proceedings of the 9th International Symposium on Artificial Intelligence and Mathematics (AI&M).

